# PReS-FINAL-2251: Influenza myositis outbreaks: clinical and laboratory findings

**DOI:** 10.1186/1546-0096-11-S2-P241

**Published:** 2013-12-05

**Authors:** SP Cardin, TP Fernandes, JG Martin, LAL Resende, CS Magalhaes

**Affiliations:** 1 Pediatrics, UNESP- São Paulo State University, Botucatu, Brazil; 2 Neurology, UNESP- São Paulo State University, Botucatu, Brazil

## Introduction

Acute myositis has epidemiologic association with Influenza, presenting with myalgia, weakness, limited mobility, high serum levels of muscle enzymes and leukopenia, in toddlers and school children, mostly in boys. It concerns the parents and puzzles physicians in emergency setting.

## Objectives

Describe epidemiology, clinical presentation and outcome of a case-series of acute viral myositis.

## Methods

Retrospective analysis of suspicious cases seen in emergency service, with follow up in rheumatology clinic, was conducted. Symptom records during respiratory infections with muscle-skeletal symptoms with investigations, including muscle enzymes (CK, LDH, AST-ALT), hematologic assessment, CRP and ESR, were analyzed at onset and follow up.

## Results

Overall 42 subjects were identified from 2000-2009, during peak flu-season and 35 (27 boys) were included. Median onset age was 7 years. Target diagnosis was reported in 89%, during first emergency visit. Observed acute respiratory symptoms, cough (31%) and coriza (23%), with fever (63%) had mean duration of 4.3 days. Muscle-skeletal symptoms were calf-pain (80%), limited walking (57%), abnormal gait (40%), muscle weakness on lower limbs (71%), all with mean duration of 3.6 days. There was a remarkable peak of muscle enzymes, CK (5,507 ± 9,180) U/l, LDH (827 ± 598) U/l and AST (199 ± 245) U/l, and also trends to leukopenia (4,59 × 10^3^ ± 1,42 × 10^3^) n/mm3. Full recovery with laboratory parameters back to normal occurred within 30 days (median). One relapse was identified with 10 months interval. Virus identification was not obtained.

## Conclusion

Typical myositis symptoms with CK peaks following flu-symptoms and a self-limited course are clues to diagnosis. CK elevation and muscle weakness indicate a myotropic activity related to B-Influenza that should be considered in outbreaks, regardless of virus identification. Awareness for this rare interesting muscle-skeletal condition is needed.

## Disclosure of interest

None declared.

